# Validating and Comparing Highly Resolved Commercial “Off the Shelf” PM Monitoring Sensors with Satellite Based Hybrid Models, for Improved Environmental Exposure Assessment

**DOI:** 10.3390/s21010063

**Published:** 2020-12-24

**Authors:** Dan Lesser, Itzhak Katra, Michael Dorman, Homero Harari, Itai Kloog

**Affiliations:** 1 Faculty of Humanities and Social Sciences, Ben Gurion University of the Negev, Beer Sheva 8410501, Israel; danless@post.bgu.ac.il (D.L.); katra@bgu.ac.il (I.K.); dorman@post.bgu.ac.il (M.D.); 2 Ichan School of Medicine at Mount Sinai, 1 Gustave L. Levy Place, New York, NY 10029-5674, USA; homero.harari@mssm.edu

**Keywords:** particulate matter, dust sensors, mobile measurements, micro-controllers, bike, Brompton bicycle

## Abstract

Particulate matter is a common health hazard, and under certain conditions, an ecological threat. While many studies were conducted in regard to air pollution and potential effects, this paper serves as a pilot scale investigation into the spatial and temporal variability of particulate matter (PM) pollution in arid urban environments in general, and Beer-Sheva, Israel as a case study. We explore the use of commercially off the shelf (COTS) sensors, which provide an economical solution for spatio-temporal measurements. We started with a comparison process against an A-grade meteorological station, where it was shown that under specific climatic conditions, a number of COTS sensors were able to produce robust agreement (mean $R^{2}=0.93,\;\mathrm{average}\;\mathrm{SD}=17.5$). The second stage examined the COTS sensors that were proven accurate in a mobile measurement campaign. Finally, data collected was compared to a validated satellite prediction model. We present how these tests and COTS sensor-kits could then be used to further explain the continuity and dispersion of particulate matter in similar areas.

## 1. Introduction

Atmospheric particular matter (PM), or aerosols, is the general term used for a mixture of solid particles and liquid droplets found in the atmosphere emanating from either natural or anthropogenic aerosols [1]. PM products are classified commonly as PM10 and PM2.5, that is particular matter with a diameter less than 10 μm and 2.5 μm, respectively. Natural sources of air pollution act as a main source of suspended matter, which includes events such as dust storms, sea spray, and crustal material. In addition, the contribution of transported dust, which is a result of soil erosion and dust storm episodes, may reach more than 60% of total PM10 in Mediterranean countries during a strong dust pollution event [2]. The main sources of anthropogenic air pollution in the form of PM were previously measured in the EU as transportation (terrestrial, naval, and aerial), residential, manufacturing industries and construction, metal production, mineral products, direct soil emissions, manure management, and other various categories [3].

In Israel, the complexity of the geo-climatic regions gives way to changing dispersal sources. For example, dust storm events in southern-Israel can reach hourly averages of 1000–5197 $\mathrm{mg}/m^{3}$ [4]. A research by Ganor et al. [5] showed an increasing trend in the total annual number of days with dust, with a slope of 0.27 days per year, concluding that the effects of dust storms on PM levels will only increase in the coming years. Krasnov et al. [6] showed that from 2009 onwards, average dust levels in Beer Sheva have risen by 30% from contributing dust storms.

There is a strong body of literature showing the adverse human health effects caused by exposure to both PM2.5 and PM10. The World Health Organization has estimated that PM2.5 contributes to approximately two million premature deaths per year, ranking it as the thirteenth leading cause of mortality worldwide [7,8,9]. Studies looking at both the long term and short term of PM exposure have shown associations with, inter alia, increased risk of myocardial infarction [9,10,11,12,13], reduced birth weights [1,14,15], cardiovascular disease [11,16,17,18,19,20], and respiratory disease [21,22,23]. Based on these findings it has been established that long-term exposure to ambient particulate matter is a major risk factor to the global burden of disease [24].

Typically, exposure to PM in epidemiological studies is assessed using measurements from air quality monitoring stations. However, in many geographical areas ground PM monitoring networks are sparse and distributed heterogeneously, which creates bias in environmental health studies [25]. Alternatively, Satellite remote sensing data can be used to assess PM in these areas. The broad spatial coverage provided by satellite based models [1,14,26,27,28] allowed us to expand exposure data far beyond the range of conventional ground monitoring networks which greatly enhances our ability to estimate subject-specific exposures. Although the use of satellite models in recent years for exposure modeling has been successfully, intra urban high resolution and real time exposure assessment methods are still needed for residence level acute health events.

There are several well established methods for measurements of high resolution robust particulate matter, such as the Tapered Element Oscillating Microbalance (TEOM). The gravimetric principle describes a quantitative determination of an analysis based on the mass of a solid. These well-established high-precision devices are all typically large, stationary, and expensive, and therefore very sparsely deployed. Typically, only a few stations will cover large urban areas [29]. The method we have used for more fine-grained measurements was laser diffraction; It relies on a device which transmits a laser beam inside the containment chamber, and based on the size and shape of the particles, the light refracts and gives different values of PM [30]. High resolution of PM measurements is important, since exposure levels have been observed to vary even in close proximity. Krasnov [31] has shown that fine grained measurements with the TSI DustTrak DRX 8534 gave a strong correlation between the spatial distribution values of PM2.5 and PM10 for the different measurement points. During dust days, however, the DustTrak’s RMSE values, when compared with the nearby station, for the averaged dust data were high, from 48 for PM10 to 25 for PM2.5, which shows that the calculated average PM spatial distributions during dust storm events were not accurate representations of the presented model, despite the relatively proximate measurements.

Given the financial and spatial limitations of these devices, researchers have started looking into portable instruments for mobile measurements based on optical light transmission. Previous studies considered the possibility of using mobile, commercial off-the-shelf (COTS) devices for a more plausible solution, giving high temporal resolution along with a wider spatial one [29,32,33]. The emergence of COTS technologies can lower the technological and financial barriers for developing countries to monitor air pollution emissions, and it can bring more citizens to participate in collecting environmental data. Access to environmental COTS sensors for measuring concentrations of carbon dioxide, nitrogen dioxide, methane, and ozone in the atmosphere can improve and democratize environmental data collection. One of the approaches that were suggested was a similar PM measurement method, using a node system of spatially distributed smart COTS sensors. Along with a connection to a wireless network at sink nodes, COTS sensors have the potential to increase spatial resolution significantly with limited effects on temporal resolution [32]. While most research are in agreement that some COTS sensors’ use in outdoor environments is possible, there is a plethora of devices that have not been vetted through scientific means. Given the multitude of devices and the lack of exposure data, it can be concluded that a proper calibration, as well as a validation, process needs to take place before they could be relied on scientifically and commercially.

The small size of the COTS sensors and their simple operation also make them very useful for mobile measurements. The choice to use bicycles as the mobile campaign’s means of transport was made given their ability to traverse longer distances without emitting additional pollutants that might intervene with accurate readings, along with their increased mobility and range in urban areas [34,35,36]. Liu [37], for instance, arranged a study where multiple public bicycles were attached with a GPS, a Bluetooth transmitter and sensing boxes that measure air quality. Once ridden, the bicycles would log pollution data and send it back to the servers once they are parked at a public bicycle station, giving a high resolution and updated map of the Air Quality Index (AQI) in the area.

Our goal in this was to evaluate, validate and compare all locally available leading COTS PM sensors. In addition, we aim at identifying a robust sensing kit for an accurate representation of PM dispersal data outdoors, specifically in a hot urban environment.

## 2. Materials and Methods

### 2.1. Study Area

**Figure 1:**
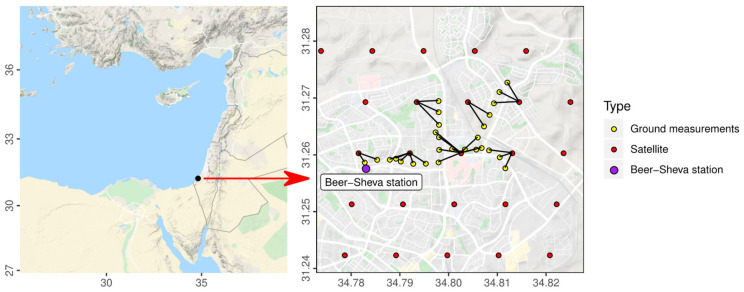
A map of the study site and the 28 mobile measurement points, along with the location of the monitoring station.

Located in the Negev desert in southern Israel, Beer Sheva has hot and dry summers, those of which are mostly attributed to daily land and sea breeze circulation, along with cold and slightly wet winters that include considerable cyclonic activity (Figure 1). The Persian trough is the main synoptic influence during the summer, which brings dry air with its northwesterly winds, as well as stable PM conditions. During winter, a calm southeasterly flow is entwined with northwesterly subtropical storms, which traverse the region on a weekly basis. During autumn, the Red Sea trough has the most effect in our region, which induces high PM levels and a stronger probability of forming dust storms. This semi-arid desert averages around 100 mm of annual rainfall, mostly during the winter season. Average annual temperature in the area is 18.3 °C, with extreme ranges that vary between seasons, as well as daily and nightly cycles. With a population of over 200,000 people, and a population density of 1700 residents for every km^2^, the effects of urban renewal along with resident’s dispersal are factors which can very much influence the susceptibility of dust events in the city, along with their epidemiological ramifications. The desert climate in Beer Sheva could simulate a global warming scenario; High levels of particulates and air pollution, given anthropogenically induced dust events; Along with the single measurement station at the center of the city. All those provided fertile ground in our attempt to calibrate and use said COTS sensors in high PM levels.

### 2.2. Environmental Data

Air quality data for preliminary comparison was collected from the single monitoring station set up by the Israel Ministry of Environmental Protection (www.sviva.gov.il) in Beer Sheva. The station is located on top of a building at the center of the city (31.25764° N, 34.78132° E), and serves as the only air quality monitoring station in the area. The station provides other meteorological data as well, including wind speed and direction, air temperature, relative humidity and more. The PM10 and PM2.5 data is recorded every 5 min by a dichotomous ambient particulate monitor (Thermo Scientific 1405-DF) for continuous direct measurements of PM, by utilizing two tapered element oscillating microbalances (TEOM) calibrated using the volumetric air flow calibrator technique (National Institute of Standards and Technology, NIST).

### 2.3. Hardware

#### 2.3.1. COTS Sensors

We included available leading COTS sensors in our measurement campaign (Table 1). The Dylos DC1700 was proven efficient in hot and dry climates (35 °C and 15%) [38] and has an internal power source. Most COTS sensors, however, excluding the Sharp GP2Y1010 (and possibly the NIDS PSX-01E) use a heating resistor to create an updraft of particles. The use of such heating has several drawbacks: First, since a current is needed to heat the resistor, the power consumption is generally higher. Second, the response time is higher, since it takes some time, usually around 30 second, until the resistor is heated up. It is noteworthy that some of these small COTS sensors (as does some professional equipment) sample the Total Suspended Particles (TSP) rather than specific size classes, so that readings are PM class equivalents. Still, for their price range they can achieve remarkable results. If used as a network of sensors [32], they are able to provide better resolution for intra-urban PM variation; Residence level exposure could be better assessed [30], and enhance station measurement’s data; and, if proven reliant, even assist in predicting and monitoring variability in extreme weather conditions in the form of dust events. Given the fact urbanized areas are also denser in population, the COTS sensors’ utility would increase given the residents’ proximity. In an attempt to explore untested instruments, two COTS sensors were selected because they have not been tested against reference instruments, the OPC-N3 and the newest Sharp GP2Y1030AU0F, along with two COTS sensors that have been used in ample scientific research [39,40,41], the DylosDC1700 and the HP Honeywell. The PurpleAir COTS sensor was considered for use in earlier stages, but was withdrawn from the study due to faulty data logging and inaccurate COTS sensor measurements.

#### 2.3.2. Kit Development

Given the fact COTS sensors have an analogue output, a micro-controller was used in the construction of the kit in order to convert those signals to digital ones, which are more statistically viable. The Arduino Uno was a natural choice due to its low power consumption and price, along with its ability to utilize coding for clearer and tidied results. A power bank was also needed given the mobile aspect of the measurement campaign; The Romoss Sense 6P was used, given its high battery capacity and a dual-USB output, which allowed us to use 2 COTS sensors at once, hence reducing the size of the air quality kit.

### 2.4. PM Measurements

In order to evaluate the COTS sensors’ validity in the field, 4 separate tests were conducted based on different criteria. The first was performed in a sterile setting inside the lab to check for similar trends and explore different variations of lag intervals. The second test tried to address the effects of wind on the reliability of the measurements. Measurements conducted outside the lab in the university campus at clear (non-dust) days. The third test was done outdoor in close proximity to a reference station, to gauge the COTS sensors’ accuracy and their sensitivity to the meteorological conditions at clear (non-dust) days. The last test was a mobile measurement campaign, to test whether or not the COTS sensors could detect spatial variations in dust concentration along specific areas in the city, while comparing them to a verified satellite model. More details on each test are given in the next sections.

#### 2.4.1. Measurements under Laboratory Conditions

**Figure 2:**
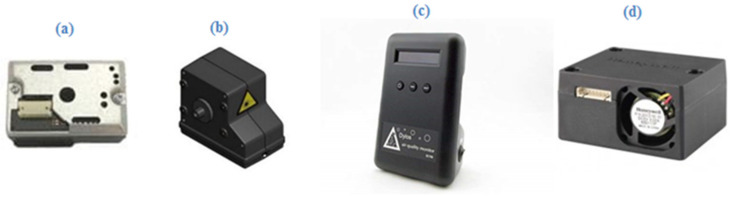
The COTS sensors that were used for the measurements: (**a**) Sharp-GP2Y1030AU0F; (**b**) OPC-N3; (**c**) Dylos DC1700; (**d**) Honeywell HPMA115S0-XXX.

The COTS sensors in Table 1 were selected based on their extended measurement ranges as well as compatibility with our micro-controller units (Figure 2). The Dylos was chosen given its built-in power source and its longevity in the field. For a better understanding on the sensors’ association to one another, preliminary measurements were made inside the laboratory to examine standard (non-dust) day’s pollution levels in an office space. Measurements were made under ambient temperature of around 25 °C, with no ventilation or external air sources, except for a small 5 V fan, given the Sharp COTS sensor’s requirement for mild airflow in order to cause particle suspension [42]. For each COTS sensor, the measurements took place at two intervals of 30 min each, in order to compile enough data for a statistical confidence analysis. The collected values were then tested against one another, to check for a correlation between the different instruments to provide a view of the ability to calibrate and/or predict dust concentration.

#### 2.4.2. Preliminary Outdoor Testing

Once the kits were assembled and functional, the COTS sensors were monitored outdoors under various wind conditions. The aim was to test its effect on the dust concentration’s measurements, and to deduce which sensors are more susceptible to bias caused by external factors, given that wind is a deciding factor in aerosol dispersion and levels [43] and may affect the validity of the COTS sensors results. The measurements were conducted in an open area, in the campus of the Ben Gurion University in Beer-Sheva under conditions of non-dust days, while wind speed was measured and evaluated at around 2.6 m/s and remained stable throughout the test for all the sensors. The COTS sensors were placed 0.5 m above the ground, their orientation remained secured and unchanging, while only their protective cardboard cover was adjusted to provide different wind exposure levels. The samples were collected for 30 min per set, which were divided into three categories according to the wind conditions. For “Full shelter” conditions, the COTS sensors were placed outdoors on a stable stone bench, but in a designated cardboard box (0.5 × 0.6 × 0.3 m). The box had an open top, and a cut up corner that allowed minor air flow at a roughly 45° exposure radius but negated the effects of wind speed and intensity on the readings. In “Partial shelter”, conditions were similar, but the protective box was cut up to provide 180° of exposure to incoming wind. Under “No shelter”, no box was used for this set of measurements, in order to provide a 360° exposure rate to test the full impact of wind speed on gathered data.

#### 2.4.3. Comparison with TEOM Data

For the next phase, for comparison purposes, the devices were tested against the PM data collected from the meteorological station in the city (Figure 1), which uses a TEOM instrument, which is a well-established reference instrument for PM measurements. This was done as a preliminary filtering process, to better understand the effects of external factors on the COTS sensors, and to discern which ones are more suitable for outdoor measurements. The COTS sensors were placed near the TEOM instrument under four different conditions: Directly next to it, on the top roof, exposed to sunlight (TE); On the bottom roof, a 3 m height difference, under shaded (BS) and not shaded (BE) conditions; and on the ground level of the building, a roughly 15 m height difference from the TEOM, which was partially exposed during the morning, and mostly shaded during the afternoon (G). The measurements used the specified cardboard box to partially shelter the COTS sensors from the wind, while the ground measurements received less wind than most, due to higher friction with the ground. Over 100 h of measurements were collected under different climatic conditions in the summer season, in order to minimize dust storm or rain effects. The testing was done during the day with an average temperature of 30 °C. Values were generated between 40 and 1300 times a minute, depending on the device. A satisfactory association between some of the COTS sensors and the station was achieved under shaded and leeward conditions (protected from direct radiation and wind). Based on the calibration’s results and their good correlation to tests in the field, the HP Honeywell and the OPC-N3 were chosen for the following measurement campaign in this study.

#### 2.4.4. Mobile Measurement Campaign by Bicycle

**Figure 3:**
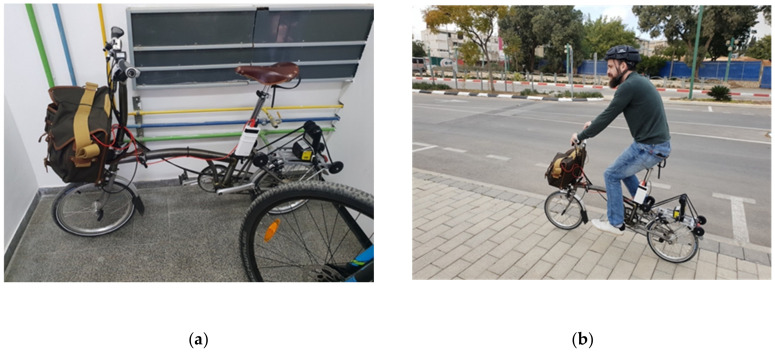
Brompton bicycles used for mobile measurements: (**a**) equipped with the COTS sensors and GPS and (**b**) in the field campaign.

The final part of the PM measurements involved an urban mobile measurement campaign, using bicycles as a means of collecting measurements. The use of bicycles was important since there is no elevation of dust or car emission levels which might affect COTS sensor readings, based on previous works that used this method [36,37,38,39]. The goal of the campaign was to check the viability of measuring PM levels at high spatial scale and to compare the results with our satellite-based model (Section 2.4.4). The bicycles used for the mobile measurements (Brompton, London, UK) were equipped with the COTS sensors and a GPS. These instruments were installed by mounts and clamps on the front of the bike (see Figure 3). Four busy streets were picked based on their location and the amount of commute they generate inside the city (Figure 1). Six measurement points were picked alongside each of these streets in 200 m intervals, for proper value separation. Along with the road points, each street was added a checkpoint that is located around 50 m away from the main road to understand the effects of transportation and our proximity to it on pollution levels. The measurements took place at each point separately, providing distinct values for each site, on 4 separate days during the summer season at an average of ~30 °C, to avoid any bias caused by rain and dust storms.

#### 2.4.5. Satellite Based PM Models

Satellite PM estimations were based on a novel satellite spatiotemporal models developed by our group [44] for predicting PM10 and PM2.5. These relied on the Moderate Resolution Imaging Spectroradiometer (MODIS) sensor on top of the Terra and Aqua satellites, based on their overpass times which differ in time (3 h apart). The instrument captures data in 36 spectral bands at varying spatial resolutions. Together, the satellites image the entire Earth every one to two days.

Specifically, the 1 × 1 km MAIAC aerosol optical depth (AOD) product from the Terra platform is available over Israel between 09:00 and 12:40 Israel Standard Time (IST), and from the Aqua platform between 11:10 and 15:00 IST. The mobile measurement times were in accordance with Terra and Aqua satellite coverage times of Beer Sheva (09:00–15:00 GMT+2) for a minimal deviation.

AOD is one of the widely-used satellite based product for PM modeling. The AOD measures light extinction at given wavelengths due to aerosol and gaseous compounds scattering absorption along the measured atmospheric column, therefore making it useful for estimating PM concentrations. The AOD product is available from several algorithms of the MODIS sensor (Deep blue, Dark target, MAIAC), on-board the two satellite platforms (Terra and Aqua). The latest developed MAIAC algorithm was recently used in various locations for PM estimation [26,45,46,47,48], due to its advantages for PM modeling including relatively high spatial resolution of 1 km, long time coverage (2000 to present time for Terra, and 2003 to present time for Aqua), and improved accuracy over bright surfaces. Daily MAIAC AOD was retrieved from Terra and Aqua Collection 6 data for the period of 2005–2015. This time period was chosen due to the higher availability of PM monitoring stations and dust events classification data. Additional details about the MAIAC product and algorithm can be found in previous publications [49]. Our models produce daily 1 × 1 km estimation for PM2.5 and PM10. The model is run in 3 stages: We start by calibrating the satellite-based aerosol optical depth (AOD) data grid-level observations to the relevant PM monitoring data collected within 1 km of an AOD reading. This is done using mixed model regressions for observed PM monitoring data that contains both fixed and day-specific random effects for the intercept, the AOD slopes, and the temperature slopes. We also incorporate additional spatial and temporal (daily) covariates as predictors in this calibration stage: Raster based spatial predictors (land-use, population density, NDVI, elevation, and road density), which were assigned to the 1 × 1 km grid cell using zonal statistics tool that allows calculating the mean raster values for each grid cell; and Meteorological temporal predictors (Air temperature, relative humidity, wind speed, rainfall, and Nitrogen Oxides concentration), which were matched to grid cells, and was averaged based on the nine closest weather stations with available meteorological variables. Dust day classification was used as well as a predictor in the satellite model. Half-hourly dust events classification data were used from the study of Yuval [50]. For each AQM PM monitor, dust events classification was attributed from the closest available PM monitor. Each daily and intra-daily time windows were classified either as a dust day (if a dust event occurred at least once during that time period) or as non-dust days (if no dust events occurred). In the second stage, we use the stage 1 model fits to estimate PM concentrations in grid cells without monitors but with available AOD measurements. Finally, estimation of continuous PM concentration levels for all grid cells in the study domain is performed by a modeling phase that estimates the PM concentrations in locations where there are no satellite based AOD observations. This stage is implemented by modeling the relationships between the estimated PM from stage 2, and the PM value from an inverse distance weighting (IDW) interpolation of PM observations from AQM stations each day, accounting for possible variability of the relationships in space using the mixed modeling approach. The exposure model was rigorously validated using standard validation methods such as “ten-fold” out of sample cross validation techniques. Model performance was excellent, with out-of-sample cross validation $R^{2}$ values of 0.84–0.87 for the PM2.5 model and 0.90–0.92 for the PM10 model. Model predictions had little bias, with cross-validated slopes of 1.00 to 1.09 for both PM fractions.

#### 2.4.6. Data Structuring and Interpolation

**Figure 4:**
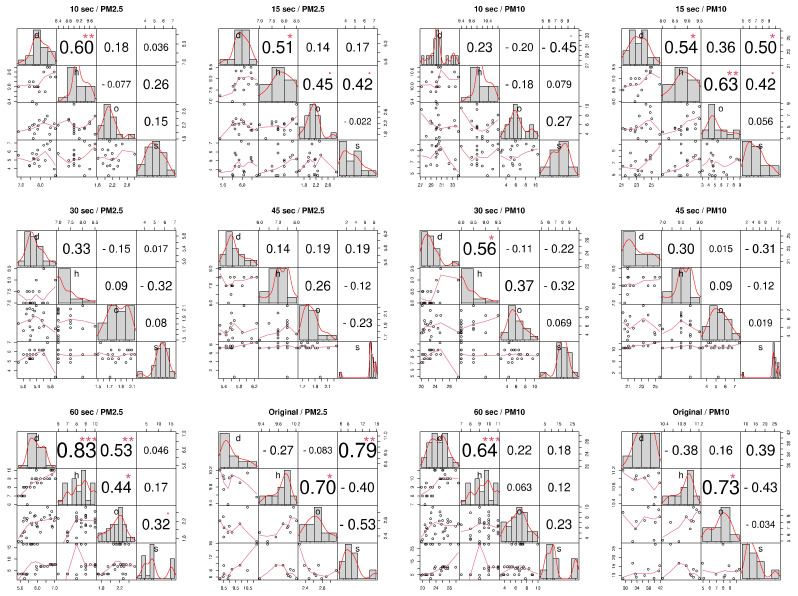
Lab measurement correlation matrix between four COTS sensors, for two particle size classes (PM.25 and PM10) and six measurements lag intervals (10, 15, 30, 45, 60 s and “original”). (H = Honeywell, D = Dylos, O = OPC, S = Sharp). Astrix denotes the signifiance level: * < 0.05, *** < 0.001, ** < 0.001.

In order to confirm the COTS sensors’ reliability, a sterile lab test was performed for a comparison of all the chosen COTS sensors. The goal of this examination was to test whether an agreement exists within the COTS sensors’ different measurement trends, to subsequently eliminate any irrelevant instruments from the experiment. After adjustments were made to the Arduino code in order to evoke different lag times between readings, Figure 4 shows the different measurements performed inside the lab under the different lag intervals, to test whether the data could be gathered in a more efficient manner, without any surplus data entries.

During our preliminary tests with the TEOM, we examined to what extent could the calibration of the COTS sensors’ be improved using the TEOM measurements, as well as surrounding meteorological conditions at the time of measurement. To do that, we fitted three types of models per PM type (2.5 and 10, respectively) and per COTS sensor (Honeywell, Sharp, Dylos, and OPC-N3):

1. Linear regression of COTS sensor PM as a function of TEOM PM
2. Linear regression of COTS sensor PM as a function of TEOM PM and four meteorological variables (temperature, relative humidity, wind speed and wind direction)
3. A random forests model of COTS sensor PM as a function of TEOM PM and the abovementioned four meteorological variables. The random forest framework was used given that its highly equipped to deal with non-linear relationships, since decision tree models in general, and random forest specifically, are able to choose by which features to split the data, with no limitation on the amount of splits. It can create a decision boundary, which is complex and non-linear.

To perform model cross-validation, and thus assess predictive ability, for each of the 24 models (2 PM types * 4 COTS sensors * 3 models), the measurements dataset was randomly split to training data (60%) and test data (40%). Each model was fitted to the training data, then model predictions on the test data were compared with the test observations. The association between predicted and observed values was evaluated by fitting a linear regression model and examining its intercept, slope, and $R^{2}$, All analyses were done in R version 4.0.2. Random Forests were fitted using the ‘randomForest’ R package.

The COTS sensor data were saved on localized SD cards during measurements and was taken to the laboratory for further analysis. After formatting and cleaning the data to be used in the analysis stage, geographic information systems (GIS) tools were used to provide a spatial layout for the mobile measurement campaign, along with a location comparison between the measurement points and the closest satellite model’s grid nodes. To enable the quantitative comparison of the different map layers, they were all produced at the same spatial scale and on the same grid under ArcGis software version 10.6.

## 3. Results

The preliminary tests and comparison assessments used Pearson’s correlation to determine whether or not a relationship exists between the COTS sensors’ PM readings and those of the TEOM. The laboratory tests showed a good correlation between the different COTS sensors (mean $R^{2}=0.75$), while some of the COTS sensors excelled outdoors as well, with varying correlations ($R^{2}=0.85-\;0.95$) when compared to the TEOM. Once the mobile campaign was finished, the results were statistically tested using regression analysis which included COTS sensor data, wind speed and direction, temperature, relative humidity and the prediction model. The values and results of the two layers were averaged and compared using R version 3.0.1+.

### 3.1. Lab Tests

**Figure 5:**
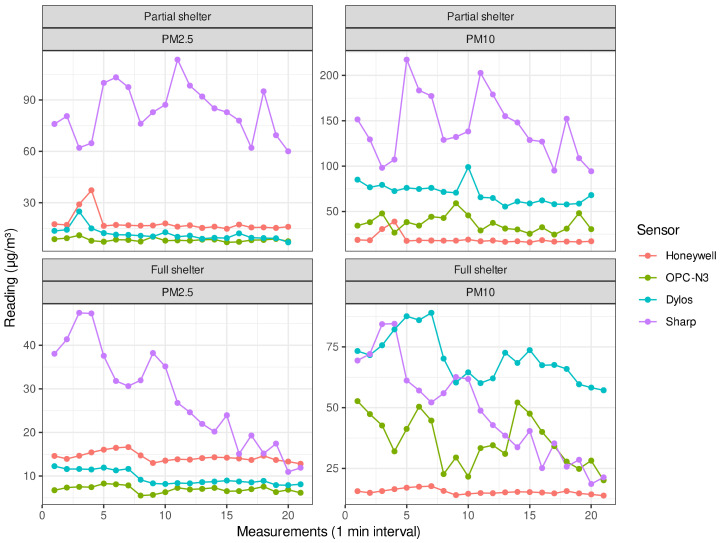
COTS Sensor readings over time under different exposure levels to wind.

Since the COTS sensors are calibrated slightly differently from one another, based on their various factory settings, it could be assumed that the results will not coincide perfectly with one another. The test was performed on 13 June 2019 at the Ben Gurion University campus (PM10: 35 μgm, PM2.5: 16 μgm, Temperature: 27 °C, Relative humidity: 45%, based on the nearby measurement station) (Figure 5). While the best result came under a 60 s delay ($R^{2}$ = 0.83 between the Honeywell and Dylos), the rest of the readings were less convincing, giving lower correlation values. Using the original lag time (ranging from 10 milliseconds to 1 s depending on the COTS sensor), however, gave a more acceptable output between the OPC and Honeywell ($R^{2}=0.7$), along with the Sharp and Dylos ($R^{2}=0.79$). Given these results, the original lag time was elected when moving forward with the research for a more reliable result. It should also be stated that the guidelines for annual mean PM levels indoors should be under 10 μg/m^3^ of PM2.5 and 20 μg/m^3^ of PM10 (WHO guidelines), which will have to be taken into consideration when moving on, by observing standard deviation units.

### 3.2. Outdoor Tests

**Figure 6:**
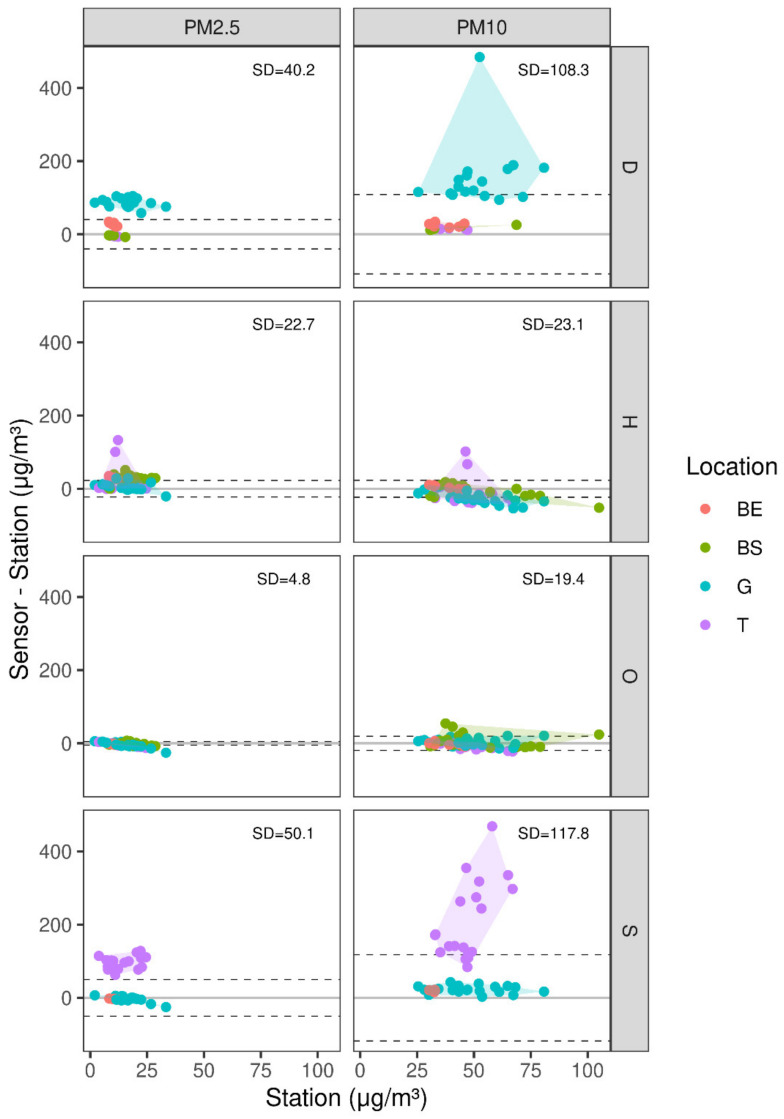
Difference between COTS sensor readings and tapered element oscillating microbalances (TEOM) station measurements, as function of TEOM measurements, per COTS sensor and particle type (D = Dylos, H = Honeywell, O = OPC-N3, S = Sharp). Dashed times are +1 SD around the y = 0 line, to emphasize the variability and bias in each case (T = Top roof, G = Ground level, BS = Bottom roof-shaded, BE = Bottom roof-exposed to sun). The shaded area represents a convex hull.

In Figure 6, wind speed was tested outdoors as the main environmental factor affecting measurement precision based on previous studies [43,51], along with previous pilot attempts to record measurements outdoors which were heavily affected by changing wind conditions. The use of the makeshift cardboard wind-protector allowed us to control the direction and intensity of this parameter, so we could control wind exposure levels to an optimal fixed degree. While the COTS sensors were completely sheltered from the wind, they showed varying correlations ($\mathrm{OPC}\;\mathrm{and}\;\mathrm{Honeywell}:\;R^{2}=\sim 0.5,\;\mathrm{Dylos}\;\mathrm{and}\;\mathrm{Honeywell}:\;R^{2}=\sim 0.7,\;\mathrm{OPC}\;\mathrm{and}\;\mathrm{Dylos}:{\;R}^{2}=\sim 0.6$). It was noted that under partial shelter (exposure of 180°, as opposed to 360° or less than 45°), the average $R^{2}$ rose to 0.8 between the COTS sensors ($\mathrm{OPC}\;\mathrm{and}\;\mathrm{Honeywell}:\;R^{2}=\sim 0.75,\;\mathrm{Dylos}\;\mathrm{and}\;\mathrm{Honeywell}:\;R^{2}=\sim 0.95,\;\mathrm{OPC}\;\mathrm{and}\;\mathrm{Dylos}:{\;R}^{2}=\sim 0.75$), confirming that wind was a crucial factor in the validity of the COTS sensors results. The Sharp COTS sensor’s sensitivity to these conditions led to an overall low correlation value ($\mathrm{average}\;R^{2}=\sim 0.4$). It could be safe to assume that while a low wind speed does not induce enough particle suspension for a proper reading, elevated wind levels could in turn jam the photo-electric measurement chamber, which could result in faulty values. While the COTS sensors’ application guide does not instruct potential ranges for bias caused by wind speed, a test that was done with the Sharp COTS sensor [51] showed a small negative wind speed trend, while most successful readings were done at a speed of 1 to 3 m/s.

### 3.3. Comparison with TEOM

Once the wind tests were completed, the COTS were taken to the monitoring station to be compared with the TEOM out in the field. The kits were placed at different locations throughout a one month measuring period during the summer (T = Top roof, G = Ground level, BS = Bottom roof-shaded, BE = Bottom roof-exposed to sun), which is considered less dusty than the preceding months. Figure 6 shows the distribution of differences between the COTS sensors’ readings and the corresponding TEOM station measurements, i.e., the COTS sensor measurement accuracy. The figure’s shaded background represents a convex hull, which divides the measurements’ locations into groups, in order to better assess their values’ divergence from one another.

The results show a steady trend with the OPC and the Honeywell COTS sensors, which have a PM2.5 SD of the deviation from station measurement of 4.8 and 22.7, and a PM10 SD of 19.4 and 23.1, respectively. The Dylos and Sharp COTS sensors, which have deviations that are closer or greater than 50 regardless of their position, were proven unreliable for our goals and were therefore omitted from the continuation of the research. The results’ division into the four measurement spots also helps in understanding the effects of wind and solar radiation exposure on the different COTS sensors; based on the readings, it was evident that the BS, followed by the BE, were the closest readings to the TEOM baseline, proving that the partial shelter exposure along with a minimal exposure to sunlight could result in better, more accurate measurements. While BS exhibited positive correlations between PM readings and wind direction, association to the TEOM was hardly noticeable for that location, along with G and T, as well. On the other hand, BS showed a high correlation between the station and the OPC, Honeywell, and Dylos COTS sensors ($R^{2}=\sim 0.93$). A similar comparison test was done when several COTS sensors were set up next to an automatic urban and rural network (AURN) station in Southampton, UK [52] for a full year. Despite finding some hardware difficulties with the Honeywell COTS sensor, the remaining COTS sensors’ uptime was well over 90%, and statistical analysis results were excellent when compared to the station’s readings (Pearson and Spearman coefficients at ~0.85 when facing east, and 0.8 when facing south or north).

**Figure 7:**
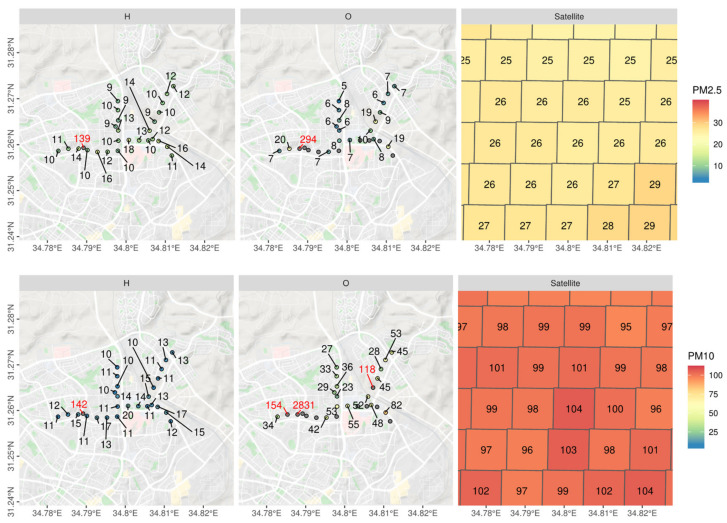
Mobile measurements campaign and spatial layout.

Models of COTS sensor PM as a function of the TEOM measurement and meteorological conditions (temperature, relative humidity, wind speed and wind direction) (Table A1 in Appendix A) showed varying degrees of predictive ability among the four COTS sensors. Dylos sensor PM could be accurately predicted using the TEOM measurements combined with the meteorological parameters $\left(\mathrm{cross}\;\mathrm{validation},\;R^{2}=0.8\right)$. However, the OPC-N3 had weaker association with the same variables ($R^{2}=0.6$). The Honeywell and Sharp sensors’ did not present the same robustness ($R^{2}=0.01{\;\mathrm{and}\;R}^{2}=0.2,\;\mathrm{respectively}$). The results may suggest that the sensitivity of Dylos and OPC-N3 sensors to the examined environmental variables is higher than that of Honeywell and Sharp. It should also be noted that the Honeywell and Sharp are exposed to external factors more than the OPC-N3 and Dylos COTS sensors, due to their less intricate design and their lack of protective exterior. The random forests model did provide a better association with the Sharp COTS sensor ($\mathrm{PM}2.5{\;R}^{2}=0.32\;\mathrm{and}\;\mathrm{PM}10{\;R}^{2}=0.42$), but the results were not conclusive enough to draw a definite effect on one another. Despite the varying results, for the purpose of the present study we focus on the accuracy of the COTS sensors’ raw measurements, i.e., the accuracy and bias of the association between COTS sensor PM and the TEOM PM measurements (Figure 7). For that reason, we consider OPC and Honeywell as the best options for outdoor measurements, given their consistency with the station’s values (see above and Figure 6).

### 3.4. Mobile Measurement Campaign

For the final stage of the experiment, the Honeywell and OPC COTS sensors were assorted into a mobile sensing kit to be used with a bicycle along pre-determined routes. The goal was to compare the COTS sensors that were proven accurate with the satellite prediction model, to validate their efficiency in field conditions. We used the previous stages’ conclusions to set up an ideal measuring platform, with partial exposure to wind and minimal exposure to sunlight, while devoiding the COTS sensors of physical vibration that may affect its accuracy.

**Figure 8:**
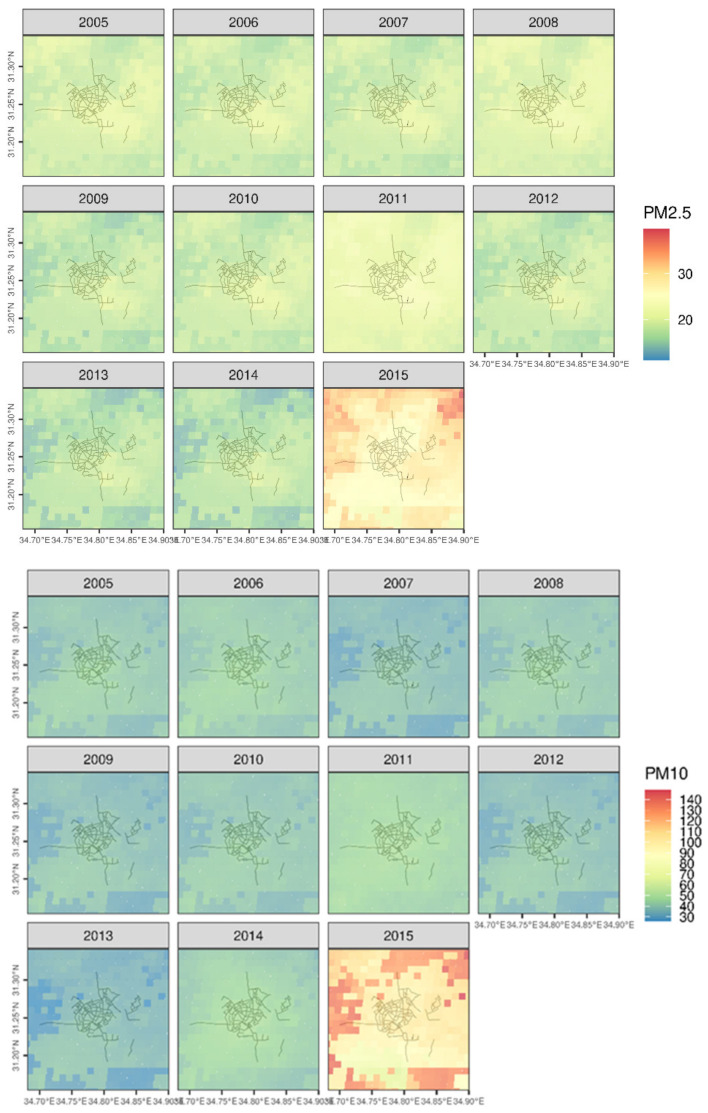
Average particulate matter (PM) per 1 km^2^ pixel in September of 2005–2015 in the city of Beer Sheva based on satellite model predictions (Top: PM2.5, bottom: PM10).

To ensure the results were not biased given specific extreme pollution conditions or an ongoing trend, we have averaged and mapped the preceding months based on the satellite readings, over a period of 10 years, as seen in Figure 8 It should be stated that PM levels during 2015 were affected by a noticeable dust storm that exceeded most of the area’s historic PM data (Average of 3372 μg/m^3^ PM10, Max; 10,280 μg/m^3^ PM10). This, in turn, has shown a tremendous rise in PM values in our analysis.

**Figure 9:**
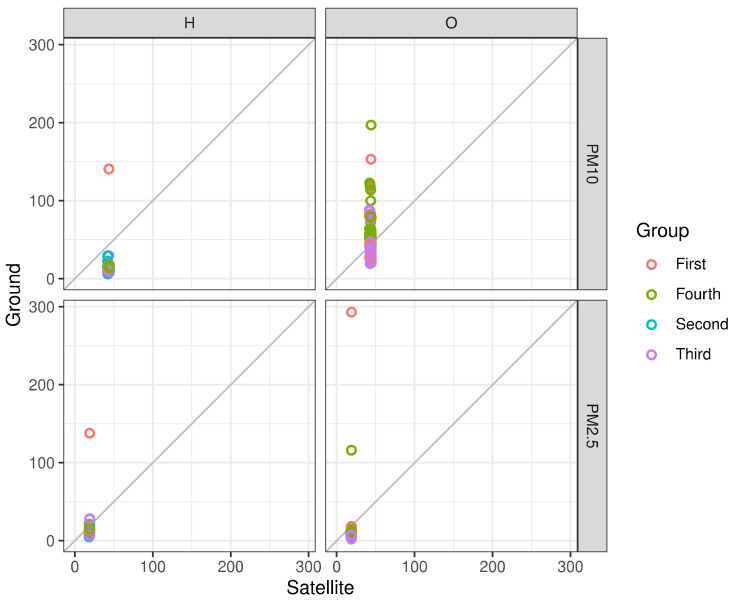
Mobile measurements comparison to satellite data, categorized by number (date of measurement).

**Figure 10:**
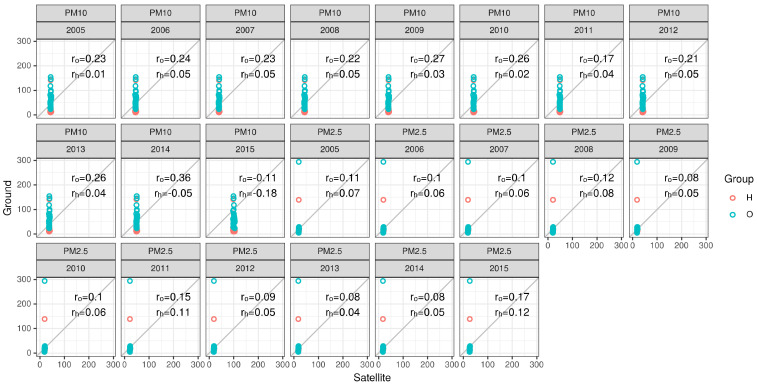
Statistical correlation between ground measurements and satellite-based PM data from September of 2005–2015.

Figure 9 shows the mobile measurement campaign, and the 4 outdoor measurement sessions compared to the satellite predictions from 2015. While the readings did not correlate perfectly with the predictions, PM2.5 readings were closer to the prediction model ($\mathrm{linear}\;\mathrm{model}\;R^{2}=\sim 0.3$) than the PM10 values ($R^{2}=\sim 0.15$). Given the fact the regression is based off of the closest neighbor method, spatially joining the closest point on the grid to its measurement counterpart, its values remain stable since the average of that period was taken into consideration. The first and fourth measurement sessions (based on date of measurements) showed a larger deviation than the other tests—this could be due to the fact that on those days (23 September 2019 and 8 October 2019, respectively), the monitoring station showed higher values of pollutants throughout the day (peaking at 80 μg/m^3^ PM10 and 25 μg/m^3^ PM2.5), which could have disrupted the COTS’ integrity given the more severe weather conditions. The issue of the COTS’ location, as well, is crucial for system identification, control, and damage detection which requires accurate measurement of the responses of the system. This is based off comparing recorded values and trend analysis to the TEOM to ensure compatibility. While the COTS showed promising results on some of the trials, the expectation was that they required additional manual calibration before operating in different environments, with emphasis on more extreme ones (dust storms, extreme heat, etc.). Figure 10 is added to enhance our understanding of the linearity of the data and shows the linear association between the both OPC-N3 and Dylos sensors and the TEOM device, along with various environmental parameters. It could be inferred from the results that relative humidity and temperature show a more linear relationship with the measured PM, mostly for the Dylos COTS sensor. There is also a clear positive, linear association based on the plots between the sensors and the TEOM device, although PM2.5 readings show a slight deviation from the station’s measurements.

**Figure 11:**
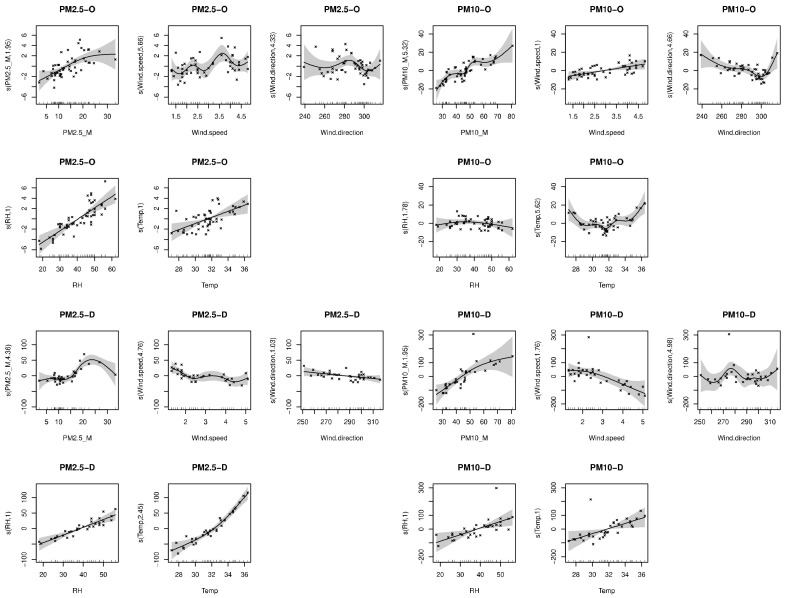
Scatter plot linearity test between the Dylos, OPC-N3 sensors and the satellite model, along with environmental parameters (PM_D = Dylos readings, PM_O = OPC-N3 readings, PM_M = TEOM readings, RH = Relative humidity).

Figure 10 shows the results of the statistical analysis performed on the ground measurements in comparison with the Satellite model PM data. It was divided per year for PM10 and PM2.5, for a better understanding of the association and in order to spot any outliers. The results showed a slight positive correlation, but not significant enough to draw a concrete conclusion. It was noticed that PM2.5 had a lower association than PM10, likely due to proximity to main roads which incurred less stable conditions for measurements, which enhances our conclusions from Figure 11 regarding the PM2.5 association to mobile measurements.

## 4. Discussion

The project set out to evaluate, validate and compare all available leading COTS PM sensors in order to identify a robust sensing kit for an accurate spatio-temporal representation of PM. We hypothesized that land cover and built environment design parameters are key levers for local control of chronic PM and that current measurement networks are insufficient to adequately capture the spatiotemporal variation of PM. Our results show that under specific conditions, some COTS sensors were reliable capturing particulate occurrences on a much smaller spatial scale. This could in turn lead to a better understanding of morbidity related to particulate matter, while providing valuable data for various treatment plans.

While the original plan was to use additional COTS sensors, such as the Novafitness SDS011 [53,54,55] and the PurpleAir [56,57,58], lack of funds and availability in the region prevented us in doing so. The choice to use the four COTS sensors we examined was based off of numerous studies [32,33,34,35,36,37,38,39,40,41,42,43,44] and was done in hopes of narrowing down the choices and select the most effective COTS sensors, both in terms of reliability and costs. One of the problems we faced was the need for a manual calibration for each of the COTS sensors separately, based on their origin and the region in which measurements are to take place. A study testing the advantages of particle COTS sensors [59] examined six performance aspect: linearity of response, precision of measurement, limit of detection, dependence on particle composition, dependence on particle size, and relative humidity and temperature influences. The standard deviations of the COTS sensors varied from 15 to 90 μg/m^3^ for a concentration range of 0–1000 μg/m^3^, which could prove problematic in higher pollution environments. The outputs of all three COTS sensors depended highly on particle composition and size, resulting in a difference that is as high as 10 times from each other in the COTS sensor outputs. These deviations prove the need for an individual calibration process for each of the COTS sensors.

The spatial resolution of the satellite models [44] was relatively large, at 1 × 1 km, compared to the smaller spatial resolution of the COTS sensors, which could have proven to be a hinderance in comparing the two methods. A recent study [60] investigated the lack of reference instruments, which created a gap in air quality information, especially in developing countries. They developed an observation-based method by combining satellite remote sensing techniques, along with low cost PM2.5 COTS sensors in California to quantify the impact of wildfires during October 2017. They have shown that a smaller, local scale for the satellite (0.1 × 0.1 km) has greater promise in utilization with ground monitors, while exhibiting difficulties in correlating them when the scale was larger. However, even at a 1 × 1 km scale, studies have shown that assimilating ground measurements in satellite based models using COTS could improve correlation value by ~0.2 [61]. Additional research is required in order to utilize 1 × 1 km models more effectively in exposure assessment, while taking into consideration different areas and pollution levels.

Along with the aforementioned limitations, such as hardware reliability and the large spatial scale, at the end of 2019, the first cases with pneumonia associated with Coronavirus (COVID-19) were reported in Wuhan, China. At some point, in a few months’ span, half the world’s population was placed on an enforced lockdown. The effects on PM levels throughout the world were noticed, and as of 9 April 2020, emissions in China have dropped by over 30% [62]. It was our hope to investigate the COTS sensors’ stability during dust episodes, but the decline in anthropogenic PM during that time would have caused bias in results that would have been compared to times with an increase in human activity, instead of a massive decline.

A study on surveying hourly PM levels, performed by Krasnov [31], discovered that a slight increase in PM10, from 38 μg/m^3^ to 45 μg/m^3^, is induced during the morning and afternoon rush hours, with a decrease after 5 p.m. This could attest to a single measurement method being a more accurate one, but on the other hand, possibly lacking in the explanation of different variables. The measurements, that were performed in the Beer Sheva and Tel Aviv areas (although the latter is less affected by the afternoon rise of natural PM10 due to Mediterranean breeze), highlight the difference between days with or without anthropogenic activity, and has shown that anthropological involvement contributes less than 15% of the measured PM10 concentration in the studied area that is close to the desert.

An outdoor evaluation performed in Salt Lake City [60] tested that theory, using two Plantower PMS 1003 and two PMS 5003 for a period of 320 days. The COTS sensors generally tracked PM2.5 concentrations compared to co-located reference air monitors (TEOM, federal gravimetric device, etc.) for optimal reliability. During elevated dust events, the COTS sensors overestimated the reference instrument by a factor of 1.47–1.89. One of the COTS sensors also exhibited a significant drift in mid-research, and continued to deteriorate through the end of the study. The COTS sensors proved satisfactory during low dust levels ($R^{2}=\sim 0.88$), which could indicate either a need for a more resilient kit to be built, or the specific COTS sensor’s lack of ability to operate in high PM environments, a fact that coincides with our tests which took place in a higher pollution level area. Additional testing is required under dust storm conditions in order to rely on the COTS sensors’ output, and to ensure the measuring chamber or internal fan are not hindered by the larger amounts of dust. The COTS optical-sensors’ refraction could also be affected by high amounts of dust, and should be considered when developing continuous measurement campaigns. Such lengthy campaigns require additional manpower and time to sustain a more continuous form of measurement, and the lack thereof within our study made us focus more intently on specific spatial and temporal points of interest.

## 5. Conclusions

The use of commercial off the shelf sensors in portraying a larger picture in patterns of particulate matter could be a significant advantage when assessing pollution rates in areas that have little to none environmental data tracking, with only a fraction of the cost of a gravimetric station. This study used the city of Beer-Sheva, which has a semi-arid hot climate and is subjected to both natural dust and anthropogenic effects, to study spatial and temporal variations of PM.

While satellite models provide a good amount of information using spectral imaging and remote sensing tools, their spatiotemporal resolution could be lacking at times given their specific overpass coverage times. Such satellite based data could be enhanced using matching ground measurements, especially in urban areas and around daily commute hotspots, by placing a network of COTS sensors for a more refined exposure profile (hourly and daily, instead of imaging-based prediction models).

The results of this paper were diverse, and showed that while the COTS sensors were relatively matched in their readings during lab conditions, different levels of exposure to wind could be a deciding factor in the values’ integrity. In situ outdoor measurements conducted next to a TEOM device showed great promise when the COTS sensors were confined to a limited exposure to radiation and wind, in which case, a proper calibration process must take place in order to take into account different areas with changing pollution levels.

Overall, it appears the compatibility between the ground measurements at high spatial resolution and satellite model (1 × 1 km) was lacking, which could also be attributed to topographic and climatic differences which are harder to take into consideration when comparing a theoretic model to actual measurements. The difference between the methods could also be explained by the discrepancy in measurement times, as dust levels have been proven to rise in the preceding years [5].

The results of this study could further help exposure assessment two folds: It will validate the use of COTS instruments as a viable PM measurement tool and enhance air pollution modeling by expanding much needed ground level data.

## Figures and Tables

**Table 1:** Description of commercially off the shelf (COTS) sensors, including their operation method, range of particulate matter concentration and approximated cost (accurate as of 13 October 2018).

COTS Sensor (Manufacturer)	Sharp-GP2Y1030AU0F	Alphanese-OPC-N3	Honeywell-HPMA115S0-XXX	Dylos-DC1700
Method	An infrared emitting diode and a phototransistor are diagonally arranged into this device, to allow it to detect the reflected light of dust in air.	OPCs provide digital outputs of PM1, PM2.5 and PM10 every second, along with histograms of the particle count for each size. Device’s flow correction improves stable readings, even in high dust environments.	utilizes a laser-based light scattering particle sensing method to detect particulates from 0.3 μm to 5 μm	Counts individual particles, gives immediate response to change in environment and provides three different history modes; minute, hour and day, up to 30 days of stored history data.
Range	0–500 μg/m^3^	0–2000 μg/m^3^	0–1000 μg/m^3^	Unspecified
Price	$12	$250	$25	$450

## Appendix A

**Table A1:** Linear and Random Forests model results between COTS sensor PM measurement and TEOM PM values, with and without meteorological factors.

Variable	Model	Slope	Intercept	R2
Honeywell-PM2.5	Linear model—TEOM	30.6388	−0.011	−0.022722584
Honeywell-PM2.5	Linear model: TEOM + Meteorological factors	27.9802	0.0552	−0.001513723
Honeywell-PM2.5	Random Forests: TEOM + Meteorological factors	28.46	0.0968	0.161438272
Honeywell-PM10	Linear model: TEOM	32.4745	−0.0534	0.019061521
Honeywell-PM10	Linear model: TEOM + Meteorological factors	29.133	0.0436	−0.028409946
Honeywell-PM10	Random Forests: TEOM + Meteorological factors	32.4729	0.0417	−0.008819204
OPC-N3-PM2.5	Linear model: TEOM	6.7076	0.3038	0.483495144
OPC-N3-PM2.5	Linear model: TEOM + Meteorological factors	2.6469	0.7821	0.57609834
OPC-N3-PM2.5	Random Forests: TEOM + Meteorological factors	6.1345	0.3937	0.554083995
OPC-N3-PM10	Linear model: TEOM	24.7203	0.4026	0.581336121
OPC-N3-PM10	Linear model: TEOM + Meteorological factors	25.6637	0.3808	0.589789448
OPC-N3-PM10	Random Forests: TEOM + Meteorological factors	34.1448	0.194	0.362786724
Sharp-PM2.5	Linear model: TEOM	54.9663	0.0014	−0.048504135
Sharp-PM2.5	Linear model: TEOM + Meteorological factors	64.0294	0.3702	0.124500294
Sharp-PM2.5	Random Forests: TEOM + Meteorological factors	50.3221	0.276	0.325243306
Sharp-PM10	Linear model: TEOM	126.2785	0.055	0.191369713
Sharp-PM10	Linear model: TEOM + Meteorological factors	159.4208	0.189	0.104123008
Sharp-PM10	Random Forests: TEOM + Meteorological factors	131.6534	0.1987	0.422302067
Dylos-PM2.5	Linear model: TEOM	52.8526	0.2487	0.140869256
Dylos-PM2.5	Linear model: TEOM + Meteorological factors	12.6777	0.9736	0.858485926
Dylos-PM2.5	Random Forests: TEOM + Meteorological factors	43.0559	0.2858	0.384341244
Dylos-PM10	Linear model: TEOM	97.8045	0.4519	0.390104326
Dylos-PM10	Linear model: TEOM + Meteorological factors	5.205	1.2015	0.828236601
Dylos-PM10	Random Forests: TEOM + Meteorological factors	103.1206	0.2891	0.661054684
